# Association between response kinetics and outcomes in relapsed/refractory multiple myeloma: analysis from TOURMALINE-MM1

**DOI:** 10.1038/s41375-018-0091-3

**Published:** 2018-03-12

**Authors:** Laurent Garderet, Jacob P. Laubach, Anne-Marie Stoppa, Parameswaran Hari, Michele Cavo, Heinz Ludwig, María-Victoria Mateos, Katarina Luptakova, Jianchang Lin, Godwin Yung, Helgi van de Velde, Deborah Berg, Philippe Moreau, Paul G. Richardson

**Affiliations:** 1 Service d’Hématologie et thérapie cellulaire, Hôpital Saint Antoine, Paris, France; 2 Hematologic Oncology, Dana-Farber Cancer Institute, Boston, MA USA; 3 Department of Hematology, Institut Paoli-Calmettes, Marseille, France; 4 Division of Hematology and Oncology, Medical College of Wisconsin, Milwaukee, WI USA; 5 Institute of Hematology and Medical Oncology “Seràgnoli” Bologna University School of Medicine, S.Orsola’s University Hospital, Bologna, Italy; 6 Wilhelminen Cancer Research Institute, Wilheminenspital, Vienna, Austria; 7 Servicio de Hematología, CIC, IBMCC (USAL-CSIC), Hospital Universitario de Salamanca, Salamanca, Spain; 8 Millennium Pharmaceuticals Inc, Cambridge, MA USA; 9 Départment d’Hématologie, University Hospital Hôtel-Dieu, Nantes, France

The association between depth of response in multiple myeloma (MM) and long-term outcomes is well recognized [1–3]. Thus, clinicians and patients are often encouraged by a rapid decrease of M-protein when treatment is initiated, and achieving ≥very-good partial response (VGPR) by 4 months of initial diagnosis has been associated with decreased mortality [4]. However, little is known about the association between response kinetics and outcomes. While some reports suggest that early responders may have compromised long-term outcomes compared with late responders [5, 6], these studies were limited, confined to frontline setting only, and based in the era prior to novel-agent availability.

Here, we evaluated progression-free survival (PFS) and duration of response (DOR) by depth of response and time to best response using data from the double-blind phase 3 TOURMALINE-MM1 trial (NCT01564537) of ixazomib-lenalidomide-dexamethasone (IRd) versus placebo-Rd in patients with relapsed/refractory MM (RRMM) [7]. The study demonstrated superior PFS with IRd versus placebo-Rd (median 20.6 versus 14.7 months, hazard ratio [HR] 0.74; *P* = 0.01) with limited additional toxicity [7], leading to the approval of ixazomib, in combination with Rd, for MM patients who had received at least one prior therapy [8, 9].

The TOURMALINE-MM1 study (NCT01564537) has been described previously [7]. Patients with RRMM were randomized 1:1 to receive IRd (*n* = 360) or placebo-Rd (*n* = 362) until disease progression (PD) or unacceptable toxicity. Response was assessed every cycle based on central laboratory results and by Independent Review Committee (IRC) evaluation [10]. The primary endpoint PFS was met at the first prespecified analysis at a median follow-up of ~15 months (median PFS, IRd versus placebo-Rd: 20.6 versus 14.7 months; HR 0.74, 95% confidence interval 0.59, 0.94, *P* = 0.01); this was the final statistical analysis of PFS [7]. A subsequent analysis for overall survival (OS) was performed after a median follow-up of ~23 months, which included a non-inferential sensitivity analysis for PFS (median PFS, IRd versus placebo-Rd: 20.0 versus 15.9 months; HR 0.82, 95% confidence interval: 0.67, 1.0) [7]. The *post-hoc* analyses reported herein are from the 23-month follow-up. At this analysis, median OS was not reached in either arm, and the trial is continuing in a double-blind, placebo-controlled fashion to allow survival data to mature.

PFS in all patients and DOR in responding patients were analyzed by depth of response, in subgroups of patients achieving stringent complete response (sCR), complete response (CR), VGPR, partial response (PR), stable disease (SD), and PD. Time-to-event curves were estimated using the Kaplan–Meier method. PFS was also analyzed in subgroups defined by time required to achieve best-confirmed response (‘time to best response’). ‘Early’ and ‘late’ responders were defined by time to best response of 0–4 and >4 months, respectively; this cut-off was chosen based on previous reports suggesting that achieving ≥VGPR by 4 months may have prognostic significance for long-term survival [4].

Since ‘late’ responders are guaranteed to have survived at least 4 months, PFS estimates may be biased in a favorable direction for late responders. To address this potential guarantee-time bias [11], duration of best response (measured from time of achieving best response to PD or death) was analyzed in early and late responders, and additional landmark and extended Cox sensitivity analyses were conducted.

Another potential bias is that achievement of a deeper response may typically take longer. Hence, late responders would be enriched for patients with deeper responses. This potential bias was addressed by conducting sensitivity analyses within individual depth of response categories. Landmark and extended Cox analyses of PFS [11] were conducted comparing early to late responders among patients achieving PR and ≥VGPR. For the landmark analyses, arbitrary cut-offs of 6 and 9 months were selected for the PR and ≥VGPR subgroups, respectively. Patients who discontinued follow-up before the cut-off timepoint were excluded. Log-rank tests were performed to test for significance at a two-sided alpha-level of 0.05 and Cox models were used to estimate and construct 95% confidence intervals for the HR comparing late to early responders.

In the extended Cox models, the period indicator for early versus late responders (0–4 months, >4 months) was replaced with a time-varying covariate that tracked whether patients had achieved PR or ≥VGPR at each timepoint. Unlike landmark analysis, an extended Cox model uses all study follow-up data and does not require the selection of arbitrary cut-offs. Together, these two approaches provide complementary and comprehensive removal of guarantee-time bias.

At the data cut-off, 676 patients across both arms had an IRC-assessed best-confirmed response: 2% sCR, 11% CR, 38% VGPR, 30% PR, 13% SD, and 6% PD. Responses deepened over time, with higher overall response rate and deeper responses seen with IRd versus placebo-Rd (Fig. 1a). Consistent with previous reports [1–3], increasing depth of response was strongly associated with improved PFS (Fig. 1b) and longer DOR across both arms (Fig. 1c) [7]. Within each response category, there was no significant difference in DOR between treatment arms; however, in the overall study population, DOR was longer with IRd versus placebo-Rd (26.0 and 21.7 months, respectively), reflecting the higher response rates and deeper responses achieved with IRd.

**Fig. 1:**
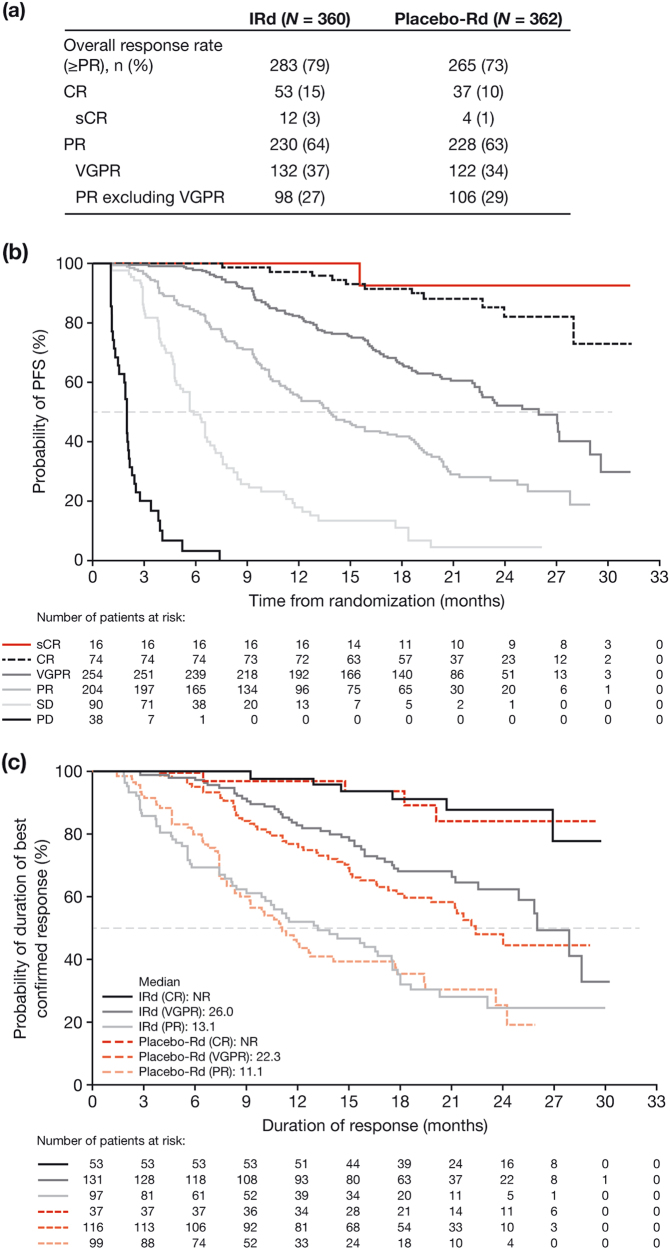
Outcomes by Independent Review Committee-assessed best-confirmed response in TOURMALINE-MM1: **a** responses seen in IRd and placebo-Rd arms; **b** progression-free survival pooled across the IRd and placebo-Rd arms based on depth of best achieved response; and **c** duration of response in the IRd and placebo-Rd arms among responders (response categories: CR, including sCR; VGPR; and PR). CR complete response, IRd ixazomib-lenalidomide-dexamethasone, NR not reached, PD progressive disease, PFS progression-free survival, PR partial response, Rd lenalidomide-dexamethasone, sCR stringent complete response, SD stable disease, VGPR very-good partial response

Analyses of outcomes by time to best response were conducted in 548 responding patients (IRd, *n* = 283; placebo-Rd, *n* = 265; patients who had SD or PD were not included). Median time to best response with IRd and placebo-Rd was 2.9 and 2.8 months, respectively, *P* > 0.05. Adjusted for best response category, patients achieved best response an average of 0.95 months earlier with IRd versus placebo-Rd (*P* = 0.02). Time to best response was 0–4 months (‘early’) or >4 months (‘late’) in 174 (61%) and 109 (39%) patients, respectively, in the IRd arm, and 159 (60%) and 106 (40%) patients in the placebo-Rd arm. There were no significant differences in baseline characteristics, including International Staging System (ISS) stage, lactate dehydrogenase (LDH) level, and high-risk cytogenetics, between early and late responders in either arm (Table S1). Early and late responders in the IRd arm received a median of 16 and 23 cycles of treatment, respectively; in the placebo-Rd arm respective medians were 15 and 23 cycles.

In both arms of the study, PFS was longer among the late versus early responders (median not reached in either arm versus 18.5 months with IRd and 14.9 months with placebo-Rd). In a sensitivity analysis to address the possibility of guarantee-time bias, the duration of the best achieved response was also longer among the late versus early responders (Figure S1).

Landmark and extended Cox analyses of PFS within the PR and ≥VGPR response categories confirmed the association between a late response and improved outcomes, while controlling for potential biases [11]. For patients achieving PR, the 6-month landmark analysis and extended Cox model showed a trend for longer PFS in late versus early responders (Fig. 2a, c). For patients achieving ≥VGPR, the 9-month landmark analysis (either treatment arm) and extended Cox model (both arms combined) showed significantly longer PFS in late versus early responders (*P* < 0.01; Fig. 2b, c).

**Fig. 2:**
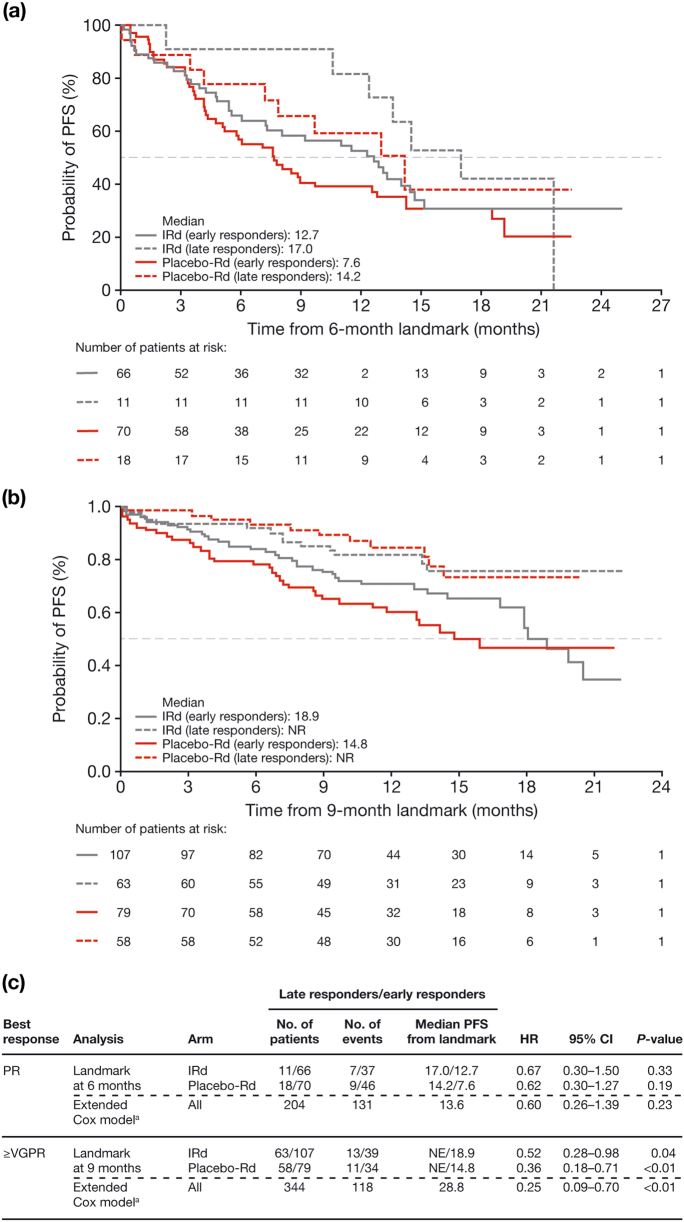
Landmark analyses of progression-free survival in the IRd and placebo-Rd arms, according to best-confirmed response: **a** from 6 months in patients achieving a partial response; **b** from 9 months in patients achieving at least a very-good partial response; and **c** summary of results and corresponding extended Cox models comparing patients who have not yet achieved best response, but who eventually will, to patients who have already achieved best response. CI confidence interval, HR hazard ratio, IRd ixazomib-lenalidomide-dexamethasone, NE not estimable, NR not reached, PFS progression-free survival, PR partial response, Rd lenalidomide-dexamethasone, VGPR very-good partial response. ^a^A Cox proportional hazards model, adjusting for treatment and a time-varying covariate tracking whether patients have achieved PR or ≥VGPR at each timepoint; patients are not classified as ‘late’ or ‘early’ responders

The overall pattern of adverse events among early and late responders (Table S2) was consistent with the primary study report [7]. Achievement of late response, and prolonged duration of therapy, did not appear to affect the safety profile of IRd or placebo-Rd.

We have confirmed the previously described [1–3] association between depth of response and PFS in patients with RRMM. However, our findings also indicate that patients achieving a late ≥VGPR had significantly longer PFS and DOR than those achieving ≥VGPR early, with a similar trend seen for patients achieving late versus early PR. One possible hypothesis explaining this phenomenon may be that patients with indolent disease and lower tumor proliferation fraction would have a slower response to therapy, but more favorable long-term outcomes [5]. Although we have not identified any significant difference in baseline characteristics, including in LDH level, ISS stage, and cytogenetic risk, that would indicate a more proliferative tumor type among the early responders, further exploration may uncover relevant biological differences between the early and late responders.

While some clinicians may be tempted to change the course of therapy if only a PR was achieved by 4 months of treatment [4], our data indicate that achievement of ≥VGPR at later than 4 months would not be detrimental to overall outcomes. A challenge and direction for future research will be to predict which patients among those who have only achieved PR by 4 months will ultimately achieve a deep response, perhaps based on their M-protein trajectory or other baseline biological variables. Those patients in PR who are receiving doublet therapy could benefit from adding a third drug to improve depth of response. However, this approach was not studied in TOURMALINE-MM1; testing this hypothesis would require additional studies. The significantly improved rates of response with IRd versus placebo-Rd in TOURMALINE-MM1 were achieved through using the triplet regimen from the start of therapy [7]. Importantly, the longer treatment duration needed to achieve best response in late responders was not associated with an additional toxicity burden. Premature discontinuation of therapy due to ‘slow response’ should therefore be avoided, and patients should be encouraged to continue treatment until progression.

## Electronic supplementary material

Supplementary material(DOCX 52 kb)
