# Supplementary material for: Association between response kinetics and outcomes in relapsed/refractory multiple myeloma: analysis from TOURMALINE-MM1
Source: Leukemia. 2018 Mar 12;32(9):2032–6. doi: 10.1038/s41375-018-0091-3 (PMC6127091; doi:10.1038/s41375-018-0091-3)
Supplement: Supplementary file 1 — Supplementary material(DOCX 52 kb) [file 41375_2018_91_MOESM1_ESM.docx]

**Association between response kinetics and outcomes in relapsed/refractory multiple myeloma: analysis from TOURMALINE-MM1**

Laurent Garderet^1^, Jacob P. Laubach^2^, Anne-Marie Stoppa^3^, Parameswaran Hari^4^, Michele Cavo^5^, Heinz Ludwig^6^, María-Victoria Mateos^7^, Katarina Luptakova^8*^, Jianchang Lin^8^, Godwin Yung^8^, Helgi van de Velde^8^, Deborah Berg^8^, Philippe Moreau^9^, Paul G. Richardson^2^

# Supplementary material

**Figure S1 (PDF)** Duration of best-achieved response in early and late responders in the IRd and placebo-Rd arms.

**Table S1 (Word)** Demographics and baseline disease characteristics in early and late responders in the IRd and placebo-Rd arms

**Table S2** **(Word)** Safety profiles of IRd and placebo-Rd in the early and late responders

**Figure S1.** Duration of best-achieved response in early and late responders in the IRd and placebo-Rd arms.

Abbreviations: IRd, ixazomib-lenalidomide-dexamethasone; NR, not reached; Rd, lenalidomide-dexamethasone.

**Table S1.** Demographics and baseline disease characteristics in early and late responders in the IRd and placebo-Rd arms

|  | *IRd* | | *Placebo-Rd* | | *P-value* |
| --- | --- | --- | --- | --- | --- |
| *Characteristic (%)* | *Early response (0–4 months) n = 174* | *Late response (> 4 months) n = 109* | *Early response (0–4 months) n = 159* | *Late response (> 4 months) n = 106* |  |
| Age ≤ 65 / 65–75 / > 75 years | 44 / 44 / 12 | 45 / 43 / 12 | 47 / 38 / 15 | 51 / 33 / 16 | 0.75 |
| Race, white / Asian / other | 87 / 7 / 5 | 88 / 7 / 5 | 80 / 13 / 7 | 88 / 7 / 5 | 0.38 |
| Male | 60 | 57 | 55 | 55 | 0.80 |
| ECOG PS 0 / 1 / 2 | 52 / 41 / 5 | 50 / 43 / 6 | 49 / 47 / 4 | 52 / 42 / 4 | 0.94 |
| ISS stage^a^, I or II / III | 88 / 12 | 87 / 13 | 87 / 13 | 94 / 6 | 0.35 |
| High- / standard-risk cytogenetics^b^ | 22 / 54 | 18 / 61 | 18 / 59 | 11 / 63 | 0.13 |
| LDH level > 250 U/L | 11 | 11 | 11 | 16 | 0.64 |
| Number of prior therapies^a^, 1 / 2–3 | 40 / 60 | 46 / 54 | 39 / 61 | 35 / 65 | 0.85 |
| Prior PI | 69 | 66 | 68 | 71 | 0.98 |
| Prior immunomodulatory drug | 52 | 54 | 50 | 57 | 0.37 |
| Relapsed / refractory / relapsed and refractory MM | 76 / 10 / 14 | 83 / 7 / 10 | 79 / 12 / 9 | 83 / 6 / 11 | 0.18 |

Abbreviations: ECOG PS, Eastern Cooperative Oncology Group performance status; FISH, fluorescence in-situ hybridization; IRd, ixazomib-lenalidomide-dexamethasone; ISS, International Staging System; LDH, lactate dehydrogenase; MM, multiple myeloma; PI, proteasome inhibitor; Rd, lenalidomide-dexamethasone.

Percentages may not total 100% due to rounding or missing data. *P*-values are based on the Cochran-Mantel-Haenszel chi-squared test for the null hypothesis that a baseline characteristic and early/late response are independent within treatment arms.
^a^Per stratification; ^b^high-risk cytogenetic abnormalities were defined as del(17p), t(4;14), and t(14;16) by FISH.

**Table S2.** Safety profiles of IRd and placebo-Rd in the early and late responders

|  | *IRd* | | *Placebo-Rd* | |
| --- | --- | --- | --- | --- |
| *AE, n (%)* | *Early response (0–4 months) n = 174* | *Late response (> 4 months) n = 112^a^* | *Early response (0–4 months) n = 159* | *Late response (> 4 months) n = 103^a^* |
| Any AE | 172 (99) | 108 (96) | 159 (100) | 103 (100) |
| Any drug-related AE | 165 (95) | 101 (90) | 147 (92) | 95 (92) |
| Any grade ≥ 3 AE | 128 (74) | 83 (74) | 108 (68) | 72 (70) |
| Any drug-related grade ≥ 3 AE | 106 (61) | 65 (58) | 82 (52) | 60 (58) |
| Any SAE | 87 (50) | 46 (41) | 73 (46) | 51 (50) |
| Any drug-related SAE | 45 (26) | 28 (25) | 37 (23) | 32 (31) |
| AE resulting in dose reduction of any drug | 103 (59) | 74 (66) | 81 (51) | 68 (66) |
| AE resulting in discontinuation of any drug | 47 (27) | 19 (17) | 30 (19) | 17 (17) |
| AE resulting in discontinuation of regimen | 38 (22) | 7 (6) | 18 (11) | 9 (9) |

Abbreviations: AE, adverse event; IRd, ixazomib-lenalidomide-dexamethasone; Rd, lenalidomide-dexamethasone; SAE, serious adverse event.
^a^Three patients in the placebo-Rd arm received a dose of ixazomib so were included in the IRd arm for safety analyses.
